# Discerning the Ambiguous Role of Missense *TTN* Variants in Inherited Arrhythmogenic Syndromes

**DOI:** 10.3390/jpm12020241

**Published:** 2022-02-08

**Authors:** Estefanía Martínez-Barrios, Georgia Sarquella-Brugada, Alexandra Pérez-Serra, Anna Fernández-Falgueras, Sergi Cesar, Mónica Coll, Marta Puigmulé, Anna Iglesias, Mireia Alcalde, Marta Vallverdú-Prats, Carles Ferrer-Costa, Bernat del Olmo, Ferran Picó, Laura López, Victoria Fiol, José Cruzalegui, Clara Hernández, Elena Arbelo, Simone Grassi, Antonio Oliva, Rocío Toro, Josep Brugada, Ramon Brugada, Oscar Campuzano

**Affiliations:** 1 Arrhythmias Unit, Hospital Sant Joan de Déu, University of Barcelona, 08950 Barcelona, Spain; estefania.martinez@sjd.es (E.M.-B.); georgia@brugada.org (G.S.-B.); sergi.cesar@gmail.com (S.C.); jvfiolramis@gmail.com (V.F.); josecarlos.cruzalegui@sjd.es (J.C.); clarahernandezcera@gmail.com (C.H.); jbrugada@clinic.cat (J.B.); 2 Medical Science Department, School of Medicine, University of Girona, 17003 Girona, Spain; 3 Cardiovascular Genetics Center, University of Girona-IDIBGI, 17190 Girona, Spain; aperez@idibgi.org (A.P.-S.); afernandez@gencardio.com (A.F.-F.); mcoll@gencardio.com (M.C.); mpuigmule@gencardio.com (M.P.); annai@brugada.org (A.I.); malcalde@gencardio.com (M.A.); mvallverdu@gencardio.com (M.V.-P.); cferrer@gencardio.com (C.F.-C.); bdelolmo@gencardio.com (B.d.O.); ferran.pico@gencardio.com (F.P.); llopez@gencardio.com (L.L.); 4 Centro de Investigación Biomédica en Red, Enfermedades Cardiovasculares (CIBERCV), 28029 Madrid, Spain; earbelo@clinic.cat; 5 Cardiology Service, Hospital Josep Trueta, University of Girona, 17007 Girona, Spain; 6 Arrhythmias Unit, Hospital Clinic, University of Barcelona-IDIBAPS, 08036 Barcelona, Spain; 7 Institute of Public Health, Section Legal Medicine, Catholic University, 20123 Rome, Italy; Simone.grassi@unicatt.it (S.G.); antonio.oliva@unicatt.it (A.O.); 8 Medicine Department, School of Medicine, University of Cadiz, 11003 Cadiz, Spain; rociotorogreen@gmail.com

**Keywords:** sudden cardiac death, inherited arrhythmogenic syndromes, *TTN*, genetics, interpretation

## Abstract

The titin gene (*TTN*) is associated with several diseases, including inherited arrhythmias. Most of these diagnoses are attributed to rare *TTN* variants encoding truncated forms, but missense variants represent a diagnostic challenge for clinical genetics. The proper interpretation of genetic data is critical for translation into the clinical setting. Notably, many *TTN* variants were classified before 2015, when the American College of Medical Genetics and Genomics (ACMG) published recommendations to accurately classify genetic variants. Our aim was to perform an exhaustive reanalysis of rare missense *TTN* variants that were classified before 2015, and that have ambiguous roles in inherited arrhythmogenic syndromes. Rare missense *TTN* variants classified before 2015 were updated following the ACMG recommendations and according to all the currently available data. Our cohort included 193 individuals definitively diagnosed with an inherited arrhythmogenic syndrome before 2015. Our analysis resulted in the reclassification of 36.8% of the missense variants from unknown to benign/likely benign. Of all the remaining variants, currently classified as of unknown significance, 38.3% showed a potential, but not confirmed, deleterious role. Most of these rare missense *TTN* variants with a suspected deleterious role were identified in patients diagnosed with hypertrophic cardiomyopathy. More than 35% of the rare missense *TTN* variants previously classified as ambiguous were reclassified as not deleterious, mainly because of improved population frequencies. Despite being inconclusive, almost 40% of the variants showed a potentially deleterious role in inherited arrhythmogenic syndromes. Our results highlight the importance of the periodical reclassification of rare missense *TTN* variants to improve genetic diagnoses and help increase the accuracy of personalized medicine.

## 1. Introduction

Inherited arrhythmogenic syndromes (IASs) are a group of genetic diseases encompassing channelopathies, principally, long QT syndrome (LQTS), short QT syndrome (SQTS), Brugada syndrome (BrS), and catecholaminergic polymorphic ventricular tachycardia (CPVT); and cardiomyopathies, mainly hypertrophic cardiomyopathy (HCM), dilated cardiomyopathy (DCM), arrhythmogenic cardiomyopathy (ACM), and left ventricular non-compacted cardiomyopathy (LVNC) [1]. IASs are characterized by malignant arrhythmias leading to ventricular fibrillation and sudden cardiac death (SCD), which is sometimes the first manifestation of disease [2]. Clinical guidelines recommend genetic testing of patients diagnosed with an IAS. If a definite pathogenic variant is identified as a cause of disease, genetic analysis should also be performed in their relatives who, despite being asymptomatic, may harbor the deleterious genetic alteration and the risk of SCD [3,4].

In 2015, the American College of Medical Genetics and Genomics (ACMG) published updated recommendations for the accurate assessment of rare variants [5], helping to define the pathogenicity of a genetic variant and proper clinical translation [6]. However, a lack of data impedes the suitable classification of many rare variants that remain of uncertain/ambiguous significance (VUS) [7]. These ambiguities leave families with inconclusive genetic diagnoses, which are not helpful in clinical decision-making [4,8]. In these cases, the ambiguous variant is disregarded in the diagnosis, and only medical and family histories are referenced for risk assessment and clinical management [5]. This situation occurs with many rare missense variants located in the *TTN* gene.

The *TTN* gene encodes the largest human protein, titin [9]. Rare variants in this 363-exon gene are associated with several musculoskeletal and cardiac diseases, including IASs [10]. Many definite deleterious *TTN* variants are truncated; few studies have reported missense variants. The roles of these rare missense *TTN* variants remain unclear and challenging to determine in IASs, mainly because of a lack of functional data. The use of the ACMG recommendations and new available data on global population frequencies may change prior classifications. The limited studies conducted in the past few years support a periodic reclassification of rare variants [11,12,13,14,15], especially if classified with an ambiguous role and not following the ACMG guidelines [16], because of the importance of understanding these variants before interpretation in a clinical setting. Here, we used currently available data to reanalyze and reclassify rare missense *TTN* variants that were previously classified as VUS in IASs.

## 2. Materials and Methods

### 2.1. Study Cohort

Our retrospective study included 193 patients with a definitive clinical diagnosis of an inherited arrhythmia, as determined following the available guidelines [17] (clinical assessment of all the patients was performed between 2011 and 2015, both years included). After comprehensive genetic analysis using a next-generation sequencing (NGS) approach, all the patients received an inconclusive genetic diagnosis explaining the origin of the disease. All the patients carried a rare missense variant in the *TTN* gene only, and the variant was classified as having an unknown/ambiguous role, according to the guidelines available at the time [18]. Suspected cases with an inconclusive clinical diagnosis were not included in the present study to avoid bias. All cases carrying a rare variant in any of the genes analyzed, classified as definitely or potentially causative of disease, were not included following the same approach. Genetic analysis was approved by the Ethics Committee of Hospital Josep Trueta (Girona, Catalonia, Spain), following the World Medical Association Declaration of Helsinki. Clinical and genetic data on all the patients were anonymized and kept confidential. Written informed consent was obtained from all the patients before genetic analysis.

### 2.2. Genetic Analysis

A resequencing custom-made panel including the most prevalent genes associated with inherited arrhythmias up until 2015 was used (*ACTC1*, *ACTN2*, *ANK2*, *CACNA1C*, *CACNB2*, *CASQ2*, *CAV3*, *CRYAB*, *CSRP3*, *DES*, *DMD*, *DSC2*, *DSG2*, *DSP*, *EMD*, *FBN1*, *GLA*, *GPD1L*, *HCN4*, *JPH2*, *JUP*, *KCNE1*, *KCNE2*, *KCNH2*, *KCNJ2*, *KCNQ1*, *LAMP2*, *LDB3*, *LMNA*, *MYBPC3*, *MYH6*, *MYH7*, *MYL2*, *MYL3*, *MYOZ2*, *PDLIM3*, *PKP2*, *PLN*, *PRKAG2*, *RYR2*, *SCN4B*, *SCN5A*, *SGCA*, *SGCB*, *SGCD*, *TAZ*, *TCAP*, *TGFB3*, *TGFBR2*, *TNNC1*, *TNNI3*, *TNNT2*, *TPM1*, *TTN*, and *VCL*). All the gene isoforms are described in Ensembl 75 (www.ensembl.org/, accessed on 5 January 2022), linked to a RefSeq code (www.ncbi.nlm.nih.gov/refseq/, accessed on 5 January 2022) or CCDS (www.ncbi.nlm.nih.gov/CCDS/, accessed on 5 January 2022). Bioinformatic analysis included adaptor and low-quality base trimming of the FASTQ files. Trimmed reads were mapped with GEM III. The output was sorted, and uniquely and properly mapped read pairs were selected. Variant calling from the cleaned BAM files was performed with SAMtools v.1.2 and an ad hoc-developed script. The final annotation steps provided information included in public databases. Identified uncommon (minor allele frequency (MAF) < 1%) genetic variants were confirmed by Sanger sequencing. The exons and exon–intron boundaries of each gene were amplified in both directions.

All sequences obtained up until 2015 were reanalyzed with updated software (SeqScape v2.7, Applied Biosystems). No new rare variants were identified in any of the analyzed genes, including the *TTN* gene. The original classification compared rare missense variants in *TTN* with DNA sequences from 200 healthy Spanish individuals (individuals not related to any index case and of the same ethnicity) as control cases, contrasted with HapMap (www.hapmap.ncbi.nlm.nih.gov/, accessed on 5 January 2022), the 1000 Genomes project (www.1000genomes.org/, accessed on 5 January 2022), and the Exome Variant Server (EVS) (www.evs.gs.washington.edu/EVS/, accessed on 5 January 2022), available in 2015. Sequence variants were described following the Human Genome Variation Society (HGVS) rules (www.hgvs.org/, accessed on 5 January 2022). Currently, all rare missense variants in *TTN* were contrasted in the Genome Aggregation Database (gnomAD) (www.gnomad.broadinstitute.org/, accessed on 5 January 2022). All rare missense *TTN* variants were also consulted in ClinGen (www.clinicalgenome.org/, accessed on 5 January 2022), VarSome (www.varsome.com/, accessed on 5 January 2022), the SCD-associated Variants Annotation Database (SVAD) (www.svad.mbc.nctu.edu.tw/, accessed on 5 January 2022), CardioClassifier (www.cardioclassifier.org/, accessed on 5 January 2022), InterVar (www.wintervar.wglab.org/, accessed on 5 January 2022), CardioVAI (www.cardiovai.engenome.com/, accessed on 5 January 2022), and CardioBoost (www.cardiodb.org/cardioboost/, accessed on 5 January 2022).

### 2.3. Data Sources

An exhaustive review of all the available data, up until December 2021, on each rare missense variant in the *TTN* gene was performed independently by three authors; afterwards, the reviews were compared and verified. Data were collected from the Human Gene Mutation Database (HGMD) (www.hgmd.org, accessed on 5 January 2022), ClinVar (www.ncbi.nlm.nih.gov/clinvar/intro/, accessed on 5 January 2022), the National Center for Biotechnology Information single-nucleotide polymorphism (SNP) database (www.ncbi.nlm.nih.gov/SNP, accessed on 5 January 2022), Index Copernicus (www.en.indexcopernicus.com), Google Scholar (www.scholar.google.es, accessed on 5 January 2022), Springer Link (www.link.springer.com, accessed on 5 January 2022), Science Direct (www.sciencedirect.com, accessed on 5 January 2022), the Excerpta Medica Database (www.elsevier.com/solutions/embase-biomedical-research, accessed on 5 January 2022), and the IEEE Xplore Digital Library (www.ieeexplore.ieee.org/Xplore/home.jsp, accessed on 5 January 2022).

#### Classification/Interpretation

All rare missense variants in *TTN* were classified following both the clinical [17] and genetic [18] recommendations available up until 2015. Currently, all the rare missense variants in *TTN* are classified following the ACMG standards and guidelines [5], including updates; the PM2 item in the ACMG classification was considered fulfilled if the MAF in the relevant population databases was ≤0.01% [19]. The vast majority of reported pathogenic variants in IASs are very rare (MAF < 0.005%) [20], consistent with all the missense *TTN* variants currently classified as likely pathogenic/pathogenic (LP/P), showing an extremely rare frequency (MAF < 0.001%) (www.gnomad.broadinstitute.org/ and www.clinicalgenome.org/, accessed on 5 January 2022). The variants classified by 2021 as VUS, supported by the available current MAF and a definite association with *TTN* disease, VUS-likely benign (VUS-LB) (MAF > 0.001%, and no definite *TTN* IAS association) and VUS-LP (no MAF or MAF < 0.001% and certain *TTN* IAS association) subgroups, were studied to clarify their potentially ambiguous role in clinical practice. Genetic data were independently evaluated and classified by five authors, specialists in the genetics of inherited arrhythmias, to avoid bias. All investigators agreed on a final classification of all the rare missense *TTN* variants included in this study.

## 3. Results

### 3.1. Cohort

Our retrospective study included 193 patients with a definitive clinical diagnosis of any IAS: 96 cases of HCM (49.74%), 30 cases of DCM (15.54%), 22 cases of LQTS (11.39%), 19 cases of BrS (9.84%), 17 cases of ARVC (8.8%), 5 cases of CPVT (2.59%), 2 cases of SQTS (1.03%), and 2 cases of LVNC (1.03%) (Table 1 and Table 2). Patients were diagnosed between 2011 and 2015, according to the clinical guidelines available at the time. The analysis of the clinical data has not changed, and IAS was definitively diagnosed.

### 3.2. Reanalysis and Reclassification

In 2015, all the patients included in our study carried only one rare missense heterozygous variant in the *TTN* gene. No patients carried any other rare alteration (including copy number variants) in any of the genes analyzed at that time. All the variants were then classified with an ambiguous role in IASs, because of a lack of available data, leaving all the cases without a conclusive genetic diagnosis.

**Figure 1:**
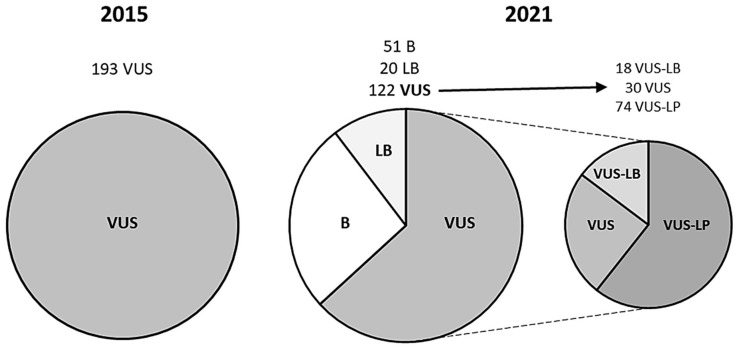
**Reclassification of rare missense *TTN* variants**. B benign, LB likely benign, LP likely pathogenic, P pathogenic, VUS variant of uncertain significance.

We performed a reanalysis that included all the available data and followed the ACMG guidelines. The reclassification showed that 71 (36.78%) rare missense *TTN* variants changed from their previous VUS classification. Twenty variants (10.36%) downgraded to LB, and 51 (26.42%) to benign (B). None of the rare missense variants in *TTN* upgraded to LP or P. Importantly, all the modifications of the first classification to LB and B were because of increased MAF from the previous classification to the present. Hence, 122 (63.22%) rare missense *TTN* variants remain classified as VUS strictly following the current ACMG guidelines (Table 1 and Table 2; Figure 1).

**Figure 2:**
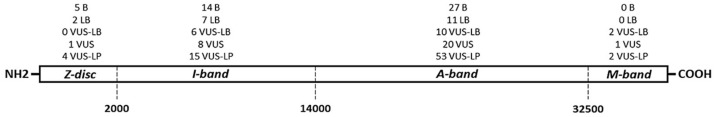
**Representative diagram of titin protein and missense variants analyzed**. Titin extends from the Z-disk of the sarcomere (N-terminus) to the M-band (C-terminus). The central part of the protein contains the I-band region (I) and the A-band region (A). B benign, LB likely benign, LP likely pathogenic, P pathogenic, VUS variant of uncertain significance.

For the detection of MAF, 57 (29.53%) variants did not show any population frequency in the present analysis, in contrast to 125 (64.76%) in 2015. In addition, 36 (0.56%) variants showed a MAF of <0.001%, but none did in 2015. All rare missense *TTN* variants without a MAF, or with a very low MAF, identified during the present analysis, remained classified as VUS because of a lack of functional data or any other additional studies, which impedes a more accurate classification following the ACMG guidelines. We developed three subgroups within the VUS category. Eighteen (9.32%) variants were reclassified as VUS-LB and 74 (38.34%) as VUS-LP. The remaining 30 (15.54%) rare missense *TTN* variants remain as VUS (Table 1 and Table 2; Figure 1). In assessing the distribution of rare missense *TTN* variants throughout the gene, we identified that the variants were mainly located in the A-band zone of the *TTN* gene, especially the variants within the VUS-LP subgroup (53 variants, 27.46%) (Figure 2).

We also analyzed by the type of IAS. In 2015, we identified 19 and 22 VUS linked with BrS and LQTS patients, respectively; the current reanalysis indicated that 10 variants in each disease (52.63% and 45.45%, respectively) remain as VUS following the ACMG guidelines. All other variants decreased in their potential to be deleterious. For CPVT, SQTS, and LVNC, the present analysis classified most variants as having decreased potential to be deleterious compared with the previous classification in 2015. In contrast, the present study classified 7 (41.17%) of the 17 VUS in ARVC patients as having an increased potential to be deleterious (VUS-LP) compared with the previous classification in 2015. In DCM patients, of the 30 variants categorized as VUS in 2015, 15 (50%) increased to VUS-LP with the current classification. Similarly, in HCM patients, 96 variants were classified as VUS in 2015, and 52 (54.16%) of these variants increased to VUS-LP following the current ACMG guidelines (Table 1 and Table 2).

## 4. Discussion

An accurate genetic diagnosis is crucial before clinical translation in IASs [3,17] Early identification of a pathogenic genetic alteration is critical in therapeutic management, reducing the risk of malignant arrhythmias and SCD [4]. The current ACMG recommendations help in obtaining an accurate genetic classification [5], but implicit stringency and a lack of sufficient scientific data lead to many rare variants remaining categorized with an ambiguous role, especially in *TTN*, a large and relatively understudied gene. A VUS does not provide conclusive data of a cause of an IAS but also cannot be disregarded [16]. Continuous genetic and clinical improvements may change a previous classification, highlighting the importance of periodically revising and clarifying the roles of VUS [21]. To our knowledge, our study is the first reanalysis focused on rare missense *TTN* variants that were previously classified as VUS. In agreement with recent studies published by our group [14,16], IAS variants that were not classified following the recommendations at the time should be immediately reclassified, especially if they were previously interpreted as VUS.

In comparison to 2015, more than 30% of rare missense *TTN* variants changed from their previous VUS classification; all decreasing their ambiguous roles to benign roles, at least in IASs. It is important to note that despite no variants being reclassified as having increased pathogenic potential in IASs, the fact that previous VUS are now classified with a benign role is critical, as these variants can be disregarded as the main cause of disease in each patient. Therefore, reducing the ambiguity of a VUS is an important advantage of a comprehensive reanalysis. From a medico-legal point of view, regular reclassification of the variants can be considered as a standard of care; being a tool for the prevention of malpractice claims: an outdated classification may lead to the risk of missed diagnosis (if the pathogenic significance of a variant is not assessed, potentially life-saving preventive interventions are not indicated) or misdiagnosis (if a benign variant is misinterpreted, useless, expensive and potentially invasive preventive interventions can be indicated) [22]. However, no interpretation should be made on its possible role in phenotype modification, and no impact on clinical management is justified according to the current clinical guidelines [3,4]. Consequently, for each patient with an IAS, rare missense *TTN* variants should be comprehensively analyzed, considering all the available data, so that they can be properly interpreted in a personalized approach [23]. It should be mandatory to update MAF in public databases, which are periodically improved and may definitively change a previously ambiguous classification, as occurs in all of our reclassified rare missense *TTN* variants. This approach, focused on population frequency, could help in a quick reclassification on the part of rare variants in *TTN*, but also in other genes associated with rare diseases. However, further studies should be performed to confirm the appropriate method for each disease/gene.

In our study, most of the rare missense *TTN* variants remain classified as VUS (63.22%). To shed light on the ambiguity implicit in VUS, subgroups were categorized according to extremely rare MAF for definite pathogenic variants in IASs [20] and the definite association of *TTN* with IASs diagnosed in each patient. Disease-specific phenotypes significantly increase the accuracy of classification and reinforce the need for clinical data in genetic diagnoses, aiding VUS interpretation [12]. Hence, of the 122 (63.21%) rare missense *TTN* variants that remain as VUS, 9.32% potentially decrease their ambiguous role (VUS-LB), 15.54% remain as VUS, and 38.74% increase their potential pathogenic role (VUS-LP). Despite all the variants remaining as VUS following the ACMG guidelines, currently available data suggest, but do not confirm, a tendency towards a benign (VUS-LB) or deleterious (VUS-LP) role in IASs. It is crucial to state that our classification should also be performed in other centers, and in large cohorts, to corroborate these VUS sub-classifications. Importantly, no clinical translation and implementation/modification of therapeutic measures should be performed according to our suggested categorization of VUS-LB or VUS-LP. Functional studies, together with a complete family segregation, may corroborate the definite role of a rare missense *TTN* variant. Our classification, despite not being conclusive, may help to select families in order to perform functional studies, especially if focused on human induced pluripotent stem cell technology. Unfortunately, for the VUS analyzed in our study, neither a complete family segregation nor a functional analysis is available. These studies will be crucial to confer a real risk of rare missense *TTN* variants in IASs [24], distinguishing real pathogenic variants from the majority of VUS that will not play a causative role in IASs. Therefore, a lack of definitive data classifies all these variants as VUS, following the current ACMG guidelines.

### 4.1. Channelopathies

First, it is important to highlight the fact that few studies have suggested a potential role of rare *TTN* variants as phenotypic modifiers in BrS, LQTS, as well as sudden arrhythmogenic death syndrome (SADS) and sudden unexplained arrhythmogenic syndrome (SUDS) [25,26,27], but no definite causative association to any inherited channelopathy is widely accepted to date. Our results are in accordance with those suggesting the non-deleterious roles of rare missense *TTN* variants in inherited channelopathies. In BrS, LQTS, and SQTS, nearly 50% of rare missense *TTN* variants currently remain as VUS following the ACMG guidelines. All other variants decrease in their deleterious roles. For CPVT, most variants demonstrate a decrease in their potential deleterious roles or remain as VUS. Therefore, clinical translation of rare missense *TTN* variants in inherited channelopathies should be taken into account, as no conclusive deleterious role has been identified to date, but the variants that remain classified as VUS following the ACMG recommendations should not be disregarded until clarifying their roles.

### 4.2. Cardiomyopathies

Rare genetic alterations in the *TTN* gene are associated with inherited cardiomyopathies, mainly DCM [10,28]. However, most reported deleterious variants are “radical” (nonsense and indels) [9,29,30], and only a few studies have examined the pathogenicity of missense *TTN* variants in inherited cardiomyopathies [31,32,33,34,35]. The clinical role of rare missense *TTN* variants remains unclear because practically all reported missense *TTN* variants have been classified as VUS following the ACMG recommendations, because of a lack of additional clinical, genetic, and functional studies concerning no radical *TTN* variants. Hence, to our knowledge, no more than 30 rare missense *TTN* variants have been definitively classified as LP or P to date. In our cohorts of DCM patients, 50% of variants previously classified as VUS increased to VUS-LP, suggesting a potential pathogenicity despite a lack of data, which impedes the ability to obtain a definite role in clinical practice. 

Patients diagnosed with HCM due to rare truncated *TTN* variants have been reported [36,37,38], but, as also occurs in other IASs, rare missense *TTN* variants have not been deeply analyzed and thus, currently remain as VUS following the ACMG recommendations. In our study, 54.16% of variants classified previously as VUS in HCM now increase to VUS-LP, suggesting potential pathogenicity. However, a lack of sufficient accurate data impedes the identification of a conclusive role that may help in clinical translation. 

For ARVC, despite some articles suggesting that *TTN* variants potentially cause disease [39,40], a definite association with ARVC or as a phenotype modifier is a current matter of debate [41,42]. In our study, most rare missense *TTN* variants were reclassified as having no deleterious role in ARVC. However, 41.17% of variants that were previously classified as VUS showed a tendency to have a potentially damaging role. This potential pathogenic predisposition is because of the extremely rare frequency in global population databases, despite which no conclusive role can be assumed following the current ACMG recommendations. We believe that potentially deleterious roles should not be disregarded *a priori* for any of these rare missense *TTN* variants, and future studies may help to clarify if they have a role in ARVC. 

In the past few years, some studies have focused on truncated *TTN* variants as potential causes of LVNC [43,44,45]. However, to our knowledge, no studies investigating the role of rare missense *TTN* variants in LVNC have been published to date. Our study showed that rare missense *TTN* variants previously classified as VUS in LVNC decrease to a benign role. Therefore, a definite association of rare missense *TTN* variants with LVNC should be confirmed, and its role in clinical translation should be interpreted with caution in each patient. 

#### Limitations

The reclassification of rare missense *TTN* variants has some limitations that should be mentioned. Firstly, the patients included in our cohort may carry additional rare variants in other genes not included in our panel. Secondly, a lack of available functional and clinical data for many rare missense variants in *TTN* impedes a more accurate interpretation, causing many variants to remain categorized with ambiguous roles in IASs. Thirdly, there is a lack of family segregation, which is critical to clarify the role of a rare variant in IASs. Despite this fact, and taking into account that variant classification is subject to inherent intra- and interlaboratory differences [46], the use of available MAF helps to discern a high percentage of the genetic background that is not damaging in IASs, as our results show. However, to strongly support our results, additional studies should be performed in other IAS cohorts carrying missense variants in the *TTN* gene. Concerning the prevalence/incidence in population, it is also important to remark that cardiovascular diseases are the main cause of deaths worldwide [47]. Focusing on the younger population, IASs are the main current cause of death. Finally, the economic cost of a comprehensive reanalysis has not been yet assessed, as well as who should assume this cost. This is a controversial point that should be deeply analyzed because each country has a unique health system.

## 5. Conclusions

Limited studies focus on the clinical and functional data of missense variants in the *TTN* gene, and this lack of data impedes a proper variant classification, leading to many variants currently being classified as VUS. Updating the genetic data and the use of the ACMG criteria results in 36.78% of rare missense variants in *TTN* that were previously classified with an ambiguous role, changing their classification to having no deleterious roles. All the identified changes were mainly because of the new available data on global frequencies. Therefore, despite no conclusive damaging role having been established for any of the analyzed variants, the 38.34% of rare missense *TTN* variants remaining as VUS could play a potentially deleterious role in IASs, mainly in HCM. Further genotype-phenotype investigations should be performed in different diseases to decide the appropriate time period for a reanalysis of rare missense variants in the *TTN* gene.

## Figures and Tables

**Table 1:** **Genetic data of rare missense *TTN* variants**. ACMG American College of Medical Genetics and Genomics, ARVC arrhythmogenic right ventricular tachycardia, B benign, BrS Brugada syndrome, ClinVar Clinical Variant, CPVT catecholaminergic polymorphic ventricular tachycardia, dbSNP Single Nucleotide Polymorphism database, DCM dilated cardiomyopathy, EVS Exome Variant Server, GnomAD Genome Aggregation Database, HCM hypertrophic cardiomyopathy, LB likely benign, LQTS long QT syndrome, LP likely pathogenic, LVNC left ventricular non-compacted, NA not available, P pathogenic, SQTS short QT syndrome, VUS variant of uncertain significance.

Disease	Exon	Nucleotide	Protein	dbSNP/ ClinVar -2015-	EVS -2015- (EA/AA/All) (%)	dbSNP/ ClinVar -2021-	GnomAD -2021- Alleles (%)	2021 Score
BrS	17	c.3100G>A	p.Val1034Met	rs142951505/VUS	0.1163/0.0/0.0769	rs142951505/B	203/250400 (0.081%)	B
BrS	37	c.8938G>A	p.Ala2980Thr	rs72647885/VUS	0.0/0.3177/0.1076	rs72647885 /LB	93/250936 (0.037%)	B
BrS	77	c.19712G>A	p.Arg6571Gln	rs372804810	0.0122/0.0/0.0084	rs372804810/VUS	10/248156 (0.004%)	VUS-LB
BrS	88	c.22940A>G	p.Asn7647Ser	rs146057575/VUS	0.0121/0.0/0.0083	rs146057575/LB	91/248780 (0.03%)	B
BrS	138	c.30010G>A	p.Val10004Ile	Novel	NA	rs996345107/VUS	NA	VUS
BrS	222	c.44643C>A	p.Asn14881Lys	Novel	NA	rs1165942763	4/246250 (0.001%)	VUS
BrS	231	c.47350G>A	p.Val15784Ile	Novel	NA	rs769334194	7/248094 (0.002%)	VUS
BrS	244	c.50303A>T	p.Asn16768Ile	rs371851242	0.0121/0.0/0.0083	rs371851242	8/248688 (0.003%)	VUS
BrS	252	c.53992G>A	p.Val17998Ile	Novel	NA	rs764777213/VUS	19/247880 (0.007%)	LB
BrS	274	c.68941G>A	p.Gly22981Ser	rs181166140	T = 0.0014/2	rs181166140/B	91/247998 (0.03%)	B
BrS	274	c.71630G>A	p.Arg23877His	Novel	NA	rs764254441/LB	10/248100 (0.004%)	VUS-LB
BrS	274	c.71887G>A	p.Glu23963Lys	Novel	NA	rs772211147/VUS	2/248206 (0.0008%)	VUS
BrS	274	c.74000T>C	p.Leu24667Pro	Novel	NA	Novel	NA	VUS
BrS	274	c.75148A>T	p.Thr25050Ser	Novel	NA	Novel	NA	VUS
BrS	274	c.75359G>A	p.Arg25120His	rs185002960	0.048/0.076/0.057	rs185002960/B	853/248882 (0.3%)	B
BrS	274	c.78119T>C	p.Leu26040Pro	Novel	NA	Novel	NA	VUS
BrS	277	c.80297A>G	p.Asp26766Gly	Novel	NA	Novel	NA	VUS
BrS	279	c.80711T>C	p.Ile26904Thr	Novel	NA	Novel	NA	VUS
BrS	306	c.98638C>T	p.Arg32880Trp	Novel	NA	rs530453291/LB	11/247068 (0.004%)	VUS-LB
CPVT	50	c.12278A>G	p.Asn4093Ser	Novel	NA	rs752924679/ VUS	15/246972 (0.006%)	LB
CPVT	81	c.20890G>A	p.Glu6964Lys	rs190192954	0.0/0.051/0.016	rs190192954/ VUS	11/248320 (0.004%)	LB
CPVT	121	c.28322C>T	p.Ala9441Val	Novel	NA	rs794729407/ VUS	NA	VUS
CPVT	172	c.33199G>A	p.Ala11067Thr	rs191699632	0.0/0.110/0.03	rs191699632/ VUS	10/156128 (0.006%)	LB
CPVT	287	c.84973A>G	p.Lys28325Glu	rs370541682	0.0/0.025/0.008	rs370541682/ VUS	7/248784 (0.002%)	VUS-LB
LQT	23	c.4090G>A	p.Ala1364Thr	Novel	NA	rs1171035485	5/250708 (0.0019%)	VUS
LQT	104	c.26753C>T	p.Thr8918Met	rs200593368/VUS	0.12/0.075/0.105	rs200593368/ LB	105/249288 (0.042%)	B
LQT	176	c.34216G>A	p.Val11406Ile	rs373881831/VUS	0.0121/0.026/0.016	rs373881831/ VUS	10/248362 (0.004%)	VUS-LB
LQT	179	c.34820T>G	p.Phe11607Cys	Novel	NA	rs575813993	12/248322 (0.004%)	VUS-LB
LQT	179	c.34952G>A	p.Ser11651Asn	Novel	NA	rs748928207	1/248508 (0.0004%)	VUS
LQT	180	c.35188G>A	p.Glu11730Lys	Novel	NA	rs778634417/ VUS	4/248512 (0.001%)	VUS
LQT	230	c.46697A>G	p.Asn15566Ser	Novel	NA	rs971483896	NA	VUS
LQT	242	c.49774G>A	p.Val16592Ile	rs200778464/VUS	0.0122/0.026/0.016	rs200778464/ VUS	23/247908 (0.009%)	B
LQT	249	c.51698G>A	p.Gly17233Asp	rs202206216	NA	rs202206216/ LB	46/248146 (0.01%)	B
LQT	252	c.53434C>A	p.Leu17812Met	rs201167216	0.0/0.262/0.083	rs201167216/ LB	67/247466 (0,02%)	B
LQT	252	c.54868A>G	p.Thr18290Ala	rs200689750/VUS	0.109/0.078/0.099	rs200689750/ LB	101/248280 (0.04%)	B
LQT	253	c.55552G>A	p.Gly18518Arg	Novel	NA	rs755518802	1/247294 (0.0004%)	VUS
LQT	261	c.57988A>C	p.Lys19330Gln	Novel	NA	rs775738687	3/245480 (0.001%)	VUS
LQT	264	c.58988G>A	p.Arg19663His	rs200971254/VUS	0.0/0.28/0.0901	rs200971254/ LB	85/247816 (0.03%)	B
LQT	265	c.59276A>G	p.Tyr19759Cys	Novel	NA	rs757701692	1/248264 (0.0004%)	VUS
LQT	272	c.61160G>C	p.Gly20387Ala	rs201381085	0.0366/0.0/0.0252	rs201381085/ LB	63/243090 (0.02%)	B
LQT	274	c.62185A>G	p.Arg20729Gly	Novel	NA	rs779474257	1/246798 (0.0004%)	VUS
LQT	274	c.67908A>T	p.Arg22636Ser	Novel	NA	Novel	NA	VUS
LQT	274	c.78614C>T	p.Thr26205Ile	Novel	NA	Novel	NA	VUS
LQT	306	c.94583C>A	p.Thr31528Asn	rs375002174	0.0121/0.0/0.0082	rs375002174/ VUS	13/247980 (0.005%)	LB
LQT	306	c.95173A>G	p.Lys31725Glu	rs72629783/VUS	0.0242/0.0/0.0166	rs72629783/ LB	54/249004 (0.02%)	B
LQT	310	c.99813T>G	p.Ser33271Arg	Novel	NA	rs776981475/ VUS	6/248908 (0.002%)	VUS-LB
SQT	2	c.208G>A	p.Val70Met	Novel	NA	rs772248060/ VUS	12/251358 (0.0047%)	LB
SQT	311	c.100160C>T	p.Thr33387Ile	Novel	NA	rs370267738/ VUS	NA	VUS
ARVC	2	c.289G>A	p.Val97Met	rs185921345	0.034/0.022/0.030	rs185921345/ B	599/251394 (0.23%)	B
ARVC	27	c.5138G>A	p.Arg1713Lys	Novel	NA	rs890589931	NA	VUS-LP
ARVC	33	c.8095T>G	p.Ser2699Ala	rs373857878	0.011/0.0/0.007	rs373857878/ VUS	2/251202 (0.00079%)	VUS-LP
ARVC	46	c.10754A>C	p.Gln3585Pro	rs375177753	0.024/0.0/0.016	rs375177753/ VUS	28/248890 (0.011%)	LB
ARVC	202	c.40051T>C	p.Tyr13351His	Novel	NA	Novel	NA	VUS-LP
ARVC	210	c.41606T>A	p.Val13869Asp	Novel	NA	rs767768313/ VUS	5/247480 (0.002%)	VUS
ARVC	211	c.41709G>T	p.Trp13903Cys	rs202094100/VUS	0.0839/0.0/0.057	rs202094100/ B	86/247226 (0.03%)	B
ARVC	217	c.43010G>A	p.Arg14337His	rs191539637	0.060/0.0/0.041	rs191539637/ B	97/247328 (0.03%)	B
ARVC	224	c.45148C>T	p.Arg15050Cys	rs201213901/VUS	0.191/0.0/0.129	rs201213901/ B	245/248616 (0.09%)	B
ARVC	257	c.56694G>T	p.Leu18898Phe	Novel	NA	rs1194410087	1/214938 (0.0004%)	VUS-LP
ARVC	257	c.56950A>G	p.Ile18984Val	rs201247592	0.0/0.133/0.041	rs201247592/ LB	27/243056 (0.01%)	B
ARVC	274	c.65321T>C	p.Ile21774Thr	Novel	NA	Novel	NA	VUS-LP
ARVC	274	c.66898A>G	p.Ile22300Val	rs72646898	0.024/0.0/0.016	rs72646898/ LB	58/247298 (0.02%)	B
ARVC	274	c.75560A>G	p.Lys25187Arg	Novel	NA	Novel	NA	VUS-LP
ARVC	278	c.80386G>A	p.Gly26796Ser	rs183013408/VUS	0.048/0.0/0.032	rs183013408/ LB	99/248324 (0.03%)	B
ARVC	282	c.81625G>A	p.Glu27209Lys	Novel	NA	Novel	NA	VUS-LP
ARVC	295	c.88720G>A	p.Val29574Ile	Novel	NA	rs779663332/ VUS	20/246836 (0.008%)	LB
DCM	15	c.2629C>A	p.Pro877Thr	Novel	NA	rs751640052/ VUS	NA	VUS-LP
DCM	28	c.6587G>A	p.Cys2196Tyr	Novel	NA	rs878854326/ VUS	1/250806 (0.00039%)	VUS-LP
DCM	38	c.9247G>A	p.Glu3083Lys	Novel	NA	Novel	NA	VUS-LP
DCM	49	c.11902G>A	p.Gly3968Arg	rs377754692	0.01/0,0/0,01	rs377754692/ VUS	7/248948 (0.0028%)	VUS
DCM	55	c.13484C>T	p.Thr4495Ile	Novel	NA	rs751087281/ VUS	NA	VUS-LP
DCM	81	c.20920A>G	p.Ser6974Gly	rs72648980/VUS	0.0/0.7105/0.2241	rs72648980/ LB	155/248460 (0.06%)	B
DCM	113	c.27659G>A	p.Arg9220Gln	Novel	NA	rs727504757/ VUS	6/174408 (0.003%)	VUS
DCM	155	c.31802A>C	p.Lys10601Thr	rs371380876	0.0123/0.0/0.0085	rs371380876/ VUS	12/248564 (0.004%)	VUS-LB
DCM	206	c.40614G>A	p.Met13538Ile	Novel	NA	rs762621076	2/245318 (0.0008%)	VUS-LP
DCM	225	c.45392G>A	p.Arg15131His	rs72646808/VUS	0.3044/0.0533/0.2256	rs72646808/ B	457/247686 (0.1%)	B
DCM	228	c.46444C>G	p.Arg15482Gly	Novel	NA	rs55734111/ VUS	NA	VUS-LP
DCM	237	c.48559C>T	p.Arg16187Cys	rs143659933	0.0121/0.0/0.0082	rs143659933	11/247830 (0.004%)	VUS-LB
DCM	242	c.49738A>G	p.Met16580Val	rs188185141	0.0603/0.0/0.0411	rs188185141/ LB	123/248042 (0.04%)	B
DCM	249	c.51830G>A	p.Arg17277His	rs201457934	0.0122/0.0/0.0083	rs201457934/ LB	26/248278 (0.01%)	B
DCM	261	c.57893G>A	p.Ser19298Asn	Novel	NA	Novel	NA	VUS-LP
DCM	261	c.57968C>T	p.Pro19323Leu	Novel	NA	rs397517662/ VUS	12/246570 (0.004%)	VUS-LB
DCM	264	c.58943G>A	p.Arg19648His	Novel	NA	Novel rs753717922	7/247984 (0.002%)	VUS
DCM	274	c.62042T>G	p.Val20681Gly	Novel	NA	Novel	NA	VUS-LP
DCM	274	c.65012T>A	p.Met21671Lys	Novel	NA	rs750298083/ VUS	8/175928 (0.004%)	VUS
DCM	274	c.73195G>A	p.Val24399Ile	Novel	NA	rs1257567608	NA	VUS-LP
DCM	274	c.73501C>G	p.Pro24501Ala	Novel	NA	rs770542451	1/246988 (0.0004%)	VUS-LP
DCM	274	c.73967A>G	p.Asn24656Ser	rs368443217/VUS	0.0122/0.0/0.0084	rs368443217/ VUS	20/243932 (0.008%)	B
DCM	274	c.74942C>T	p.Ala24981Val	Novel	NA	rs749950083	NA	VUS-LP
DCM	274	c.78758G>C	p.Gly26253Ala	Novel	NA	Novel	NA	VUS-LP
DCM	277	c.80081T>C	p.Val26694Ala	Novel	NA	rs1053653300/VUS	2/248614 (0.0008%)	VUS-LP
DCM	283	c.81985C>T	p.Leu27329Phe	rs180798672	A=0.0005/1	rs180798672/ VUS	5/248826 (0.002%)	VUS
DCM	291	c.87711C>A	p.Phe29237Leu	Novel	NA	rs587780983/ VUS	10/248118 (0.004%)	VUS-LB
DCM	306	c.95905C>T	p.Arg31969Trp	Novel	NA	rs753957126	14/249090 (0.005%)	VUS-LB
DCM	306	c.96068G>A	p.Arg32023Gln	Novel	NA	rs778021095/ VUS	3/248866 (0.001%)	VUS-LP
DCM	306	c.98408A>G	p.Asn32803Ser	Novel	NA	rs1200663369	1/248986 (0.0004%)	VUS-LP
HCM	5	c.758C>T	p.Thr253Ile	Novel	NA	rs945095401	NA	VUS-LP
HCM	15	c.2611G>T	p.Val871Leu	rs72647861	NA	rs72647861/ VUS	18/251176 (0.0071%)	LB
HCM	19	c.3241G>A	p.Ala1081Thr	rs55914517/VUS	0.1047/0.0454/0.0846	rs55914517/ LB	168/251122 (0.069%)	B
HCM	21	c.3716A>G	p.Tyr1239Cys	Novel	NA	rs794729569	2/250772 (0.0007%)	VUS-LP
HCM	24	c.4396T>C	p.Phe1466Leu	rs151310601	0.1279/0.0681/0.1076	rs151310601/ LB	173/250944 (0.068%)	B
HCM	27	c.5740G>A	p.Ala1914Thr	rs118161093	T=0.0009/2	rs118161093/ LB	35/251134 (0.013%)	B
HCM	37	c.8920A>G	p.Met2974Val	Novel	NA	rs993689796	NA	VUS-LP
HCM	43	c.10162C>T	p.Arg3388Trp	Novel	NA	rs758680640/ LB	5/250482 (0.0019%)	VUS
HCM	49	c.12014A>G	p.Gln4005Arg	Novel	NA	Novel	NA	VUS-LP
HCM	51	c.12581A>G	p.Gln4194Arg	rs190636272/VUS	0.0964/0.0254/0.0736	rs190636272/ LB	147/247774 (0.059%)	B
HCM	52	c.12814G>T	p.Ala4272Ser	rs72648940/VUS	0.1339/0.0/0.092	rs72648940/ LB	132/248410 (0.053%)	B
HCM	53	c.13075C>G	p.Leu4359Val	Novel	NA	Novel	NA	VUS-LP
HCM	62	c.15418C>A	p.Pro5140Thr	rs72648953/VUS	0.2188/0.0267/0.1586	rs72648953/ B	738/242524 (0.3%)	B
HCM	66	c.16609G>A	p.Glu5537Lys	rs72648958/VUS	0.1461/0.0/0.1003	rs72648958/ LB	248/245308 (0.1%)	B
HCM	77	c.19723G>C	p.Glu6575Gln	rs201420077	0.1097/0.0/0.0755	rs201420077/ LB	45/248270 (0.018%)	B
HCM	78	c.19943T>G	p.Ile6648Arg	rs201181445	0.0122/0.0/0.0084	rs201181445	4/244710 (0.001%)	VUS-LP
HCM	78	c.20030A>G	p.Lys6677Arg	Novel	NA	Novel	NA	VUS-LP
HCM	80	c.20375C>T	p.Ser6792Leu	rs200598509/VUS	0.0242/0.0/0.0165	rs200598509/ VUS	18/248776 (0.0072%)	LB
HCM	81	c.20887G>A	p.Asp6963Asn	Novel	NA	rs756813056/ VUS	8/248344 (0.003%)	VUS-LB
HCM	82	c.21220G>A	p.Val7074Ile	rs200103997	0.0847/0.0256/0.0658	rs200103997/ LB	146/248762 (0.058%)	B
HCM	88	c.22762A>G	p.Ile7588Val	rs72648989	0.0981/0.0/0.0678	rs72648989/ LB	190/245890 (0.07%)	B
HCM	89	c.23203A>C	p.Asn7735His	rs376982715/VUS	0.012/0.0/0.0082	rs376982715/ LB	34/248706 (0.013%)	B
HCM	96	c.25117G>A	p.Glu8373Lys	rs144025230	T=0.0009/2	rs144025230/ VUS	10/249144 (0.004%)	LB
HCM	98	c.25582G>A	p.Val8528Met	Novel	NA	rs563073635/ LB	14/249214 (0.0056%)	LB
HCM	114	c.27707A>C	p.His9236Pro	Novel	NA	Novel	NA	VUS-LP
HCM	119	c.28179A>C	p.Glu9393Asp	Novel	NA	Novel	NA	VUS-LP
HCM	127	c.28888G>A	p.Glu9630Lys	Novel	NA	Novel	NA	VUS-LP
HCM	138	c.29974C>T	p.Leu9992Phe	Novel	NA	rs967680270	NA	VUS-LP
HCM	138	c.30040G>A	p.Glu10014Lys	Novel	NA	rs778130826	1/240058 (0.0004%)	VUS-LP
HCM	152	c.31502C>T	p.Pro10501Leu	Novel	NA	Novel	NA	VUS-LP
HCM	153	c.46630G>C	p.Ala15544Pro	Novel	NA	Novel	NA	VUS-LP
HCM	184	c.35996G>C	p.Arg11999Thr	Novel	NA	rs555652524	3/240444 (0.001%)	VUS-LP
HCM	197	c.38683G>A	p.Gly12895Arg	rs200042932/VUS	0.0365/0.0271/0.0336	rs200042932/ LB	72/246698 (0.02%)	B
HCM	204	c.40364C>T	p.Ser13455Phe	Novel	NA	rs949595987	NA	VUS-LP
HCM	210	c.41471C>A	p.Ala13824Glu	Novel	NA	rs750310775/ VUS	1/247347 (0.0004%)	VUS-LP
HCM	227	c.45932T>C	p.Ile15311Thr	Novel	NA	rs760585965	4/246104 (0.001%)	VUS-LP
HCM	230	c.46801C>A	p.Pro15601Thr	Novel	NA	Novel	NA	VUS-LP
HCM	230	c.47036T>C	p.Met15679Thr	rs200585270	NA	rs200585270/ LB	80/248418 (0.03%)	B
HCM	234	c.47651C>G	p.Ser15884Cys	Novel	NA	rs567515550	3/244826 (0.001%)	VUS-LP
HCM	240	c.49369G>A	p.Val16457Ile	rs181957743	T=0.0009/2	rs181957743/ LB	45/244382 (0.01%)	B
HCM	245	c.50516C>G	p.Thr16839Ser	Novel	NA	Novel	NA	VUS-LP
HCM	245	c.50693G>C	p.Gly16898Ala	rs201922910	0.0361/0.0/0.0245	rs201922910/ LB	38/248508 (0.01%)	B
HCM	249	c.51661G>A	p.Asp17221Asn	Novel	NA	rs1352997069/VUS	9/247586 (0.003%)	VUS-LB
HCM	250	c.51953T>G	p.Val17318Gly	Novel	NA	rs755949982/ VUS	13/243972 (0.005%)	LB
HCM	251	c.52301A>G	p.Asp17434Gly	rs199512049/VUS	0.1099/0.0/0.0756	rs199512049/ LB	149/248058 (0.06%)	B
HCM	252	c.52562T>C	p.Val17521Ala	Novel	NA	rs886039085/ VUS	4/227726 (0.001%)	VUS-LP
HCM	252	c.53272G>A	p.Ala17758Thr	rs370995867	0.0121/0.0/0.0083	rs370995867/ LB	30/246926 (0.01%)	B
HCM	252	c.55343G>C	p.Ser18448Thr	Novel	NA	rs1169239147/VUS	NA	VUS-LP
HCM	254	c.55885A>G	p.Ile18629Val	rs72646855/VUS	0.0726/0.0258/0.0577	rs72646855/ LB	144/247742 (0.05%)	B
HCM	262	c.58382G>A	p.Arg19461His	rs72646868/VUS	0.0122/0.0/0.0083	rs72646868/ VUS	13/247276 (0.005%)	LB
HCM	263	c.58522T>A	p.Trp19508Arg	Novel	NA	Novel	NA	VUS-LP
HCM	264	c.58946T>G	p.Phe19649Cys	Novel	NA	rs764330098/ VUS	2/248030 (0.0008%)	VUS-LP
HCM	267	c.59659G>A	p.Val19887Ile	Novel	NA	rs759848349	4/246976 (0.001%)	VUS-LP
HCM	267	c.59894C>T	p.Thr19965Ile	Novel	NA	Novel	NA	VUS-LP
HCM	268	c.60393G>C	p.Gln20131His	Novel	NA	rs727504520/ VUS	13/248510 (0.005%)	LB
HCM	269	c.60596C>T	p.Pro20199Leu	Novel	NA	rs775953148/ VUS	7/246890 (0.002%)	VUS-LB
HCM	274	c.62924A>T	p.Asp20975Val	Novel	NA	Novel	NA	VUS-LP
HCM	274	c.63604G>A	p.Asp21202Asn	Novel	NA	rs747260758	14/246618 (0.005%)	LB
HCM	274	c.63644G>A	p.Arg21215His	rs145504744	T=0.0009/2	rs145504744/ VUS	11/246464 (0.004%)	VUS
HCM	274	c.64831G>A	p.Val21611Ile	Novel	NA	rs1400549364	1/168286 (0.0005%)	VUS-LP
HCM	274	c.68123C>A	p.Thr22708Asn	Novel	NA	Novel	NA	VUS-LP
HCM	274	c.69946G>A	p.Glu23316Lys	Novel	NA	rs747699837	4/248188 (0.001%)	VUS-LP
HCM	274	c.70579C>G	p.Pro23527Ala	Novel	NA	rs912485960	1/248792 (0.0004%)	VUS-LP
HCM	274	c.70972G>C	p.Glu23658Gln	Novel	NA	rs754878387/ LB	18/248186 (0.007%)	LB
HCM	274	c.71161C>T	p.Pro23721Ser	Novel	NA	rs1030641242	NA	VUS-LP
HCM	274	c.72422T>C	p.Ile24141Thr	Novel	NA	rs757763969	1/248214 (0.0004%)	VUS-LP
HCM	274	c.72559T>C	p.Phe24187Leu	rs200181804/VUS	0.0243/0.0/0.0166	rs200181804/ LB	36/248728 (0.01%)	B
HCM	274	c.72722G>A	p.Gly24241Asp	Novel	NA	rs532522359	7/248186 (0.002%)	VUS-LB
HCM	274	c.72997A>G	p.Ile24333Val	rs201562505	0.1/0/0.09	rs201562505/ B	403/248414 (0.1%)	B
HCM	274	c.73675A>C	p.Ile24559Leu	Novel	NA	rs1325322358	1/245806 (0.0004%)	VUS-LP
HCM	274	c.74571G>C	p.Gln24857His	Novel	NA	Novel	NA	VUS-LP
HCM	274	c.75283T>G	p.Ser25095Ala	Novel	NA	Novel	NA	VUS-LP
HCM	274	c.75465G>T	p.Glu25155Asp	Novel	NA	Novel	NA	VUS-LP
HCM	274	c.76732G>A	p.Val25578Ile	rs371366196	0.0243/0.0/0.0166	rs371366196/ VUS	10/245028 (0.004%)	LB
HCM	274	c.77758G>A	p.Val25920Ile	rs377264123	0.012/0.0/0.0082	rs377264123/ LB	17/248104 (0.006%)	LB
HCM	282	c.81646G>T	p.Val27216Phe	Novel	NA	Novel	NA	VUS-LP
HCM	286	c.84247C>T	p.Pro28083Ser	Novel	NA	rs927540266	NA	VUS-LP
HCM	287	c.85076T>A	p.Ile28359Lys	Novel	NA	rs531432790/ LB	157/248510 (0.06%)	B
HCM	287	c.85081G>A	p.Ala28361Thr	Novel	NA	rs1192451781/VUS	1/248372 (0.0004%)	VUS-LP
HCM	287	c.85301G>T	p.Ser28434Ile	rs180975448	0.0846/0.0/0.0575	rs180975448/ LB	159/248444 (0.06%)	B
HCM	287	c.85877A>G	p.Tyr28626Cys	Novel	NA	rs543223589/ VUS	12/248882 (0.004%)	LB
HCM	288	c.86578C>T	p.Arg28860Cys	Novel	NA	rs190282707/ VUS	6/248622 (0.002%)	VUS-LB
HCM	291	c.87632T>C	p.Ile29211Thr	Novel	NA	rs1460359915/VUS	1/248674 (0.0004%)	VUS-LP
HCM	292	c.87995T>A	p.Phe29332Tyr	Novel	NA	Novel	NA	VUS-LP
HCM	295	c.89159C>G	p.Pro29720Arg	Novel	NA	Novel	NA	VUS-LP
HCM	296	c.89236A>G	p.Lys29746Glu	Novel	NA	Novel	NA	VUS-LP
HCM	296	c.89395C>T	p.Arg29799Cys	rs202064385/VUS	0.0243/0.0/0.0166	rs202064385/ LB	110/248626 (0.04%)	B
HCM	299	c.90118A>G	p.Arg30040Gly	Novel	NA	Novel	NA	VUS-LP
HCM	303	c.91623T>A	p.Asp30541Glu	Novel	NA	Novel	NA	VUS-LP
HCM	304	c.92444T>C	p.Val30815Ala	Novel	NA	rs1237202129	NA	VUS-LP
HCM	306	c.93637A>G	p.Ile31213Val	Novel	NA	rs1029128193	NA	VUS-LP
HCM	306	c.93829T>C	p.Tyr31277His	Novel	NA	rs1227515280/VUS	1/248712 (0.0004%)	VUS-LP
HCM	306	c.94693A>C	p.Ile31565Leu	Novel	NA	rs1328046092	1/249066 (0.0004%)	VUS-LP
HCM	306	c.95708G>A	p.Arg31903Gln	rs149391616	T = 0.0028/5	rs149391616/ LB	80/248208 (0.03%)	B
HCM	306	c.96710G>A	p.Arg32237Gln	rs115150240/VUS	0.0121/0.0/0.0083	rs115150240/ LB	33/247298 (0.013%)	B
HCM	308	c.99357G>C	p.Lys33119Asn	Novel	NA	rs113190638	1/247918 (0.0004%)	VUS-LP
LVNC	62	c.15430G>A	p.Val5144Ile	Novel	NA	rs550617268/ LB	12/246042 (0.0048%)	VUS-LB
LVNC	274	c.64442T>C	p.Leu21481Pro	rs56399205	0.0975/0.0266/0.0752	rs56399205/ B	140/248224 (0.05%)	B

**Table 2:** **Ratio changes and disease**. Rare missense *TTN* variants (percentage). ARVC arrhythmogenic right ventricular tachycardia, B benign, BrS Brugada syndrome, CPVT catecholaminergic polymorphic ventricular tachycardia, DCM dilated cardiomyopathy, HCM hypertrophic cardiomyopathy, LB likely benign, LQTS long QT syndrome, LP likely pathogenic, LVNC left ventricular non-compacted, NA not available, P pathogenic, SQTS short QT syndrome, VUS variant of uncertain significance.

	2015	2021				
	VUS	B	LB	VUS-LB	VUS	VUS-LP
**BrS**	19 (9.84%)	5 (2.59%)	1 (0.51%)	3 (1.55%)	10 (5.18%)	0
**CPVT**	5 (2.59%)	0	3 (1.55%)	1 (0.51%)	1 (0.51%)	0
**LQTS**	22 (11.39%)	8 (4.14%)	1 (0.51%)	3 (1.55%)	10 (5.18%)	0
**SQTS**	2 (1.03%)	0	1 (0.51%)	0	1 (0.51%)	0
**ARVC**	17 (8.8%)	7 (3.62%)	2 (1.03%)	0	1 (0.51%)	7 (3.62%)
**DCM**	30 (15.54%)	5 (2.59%)	0	5 (2.59%)	5 (2.59%)	15 (7.77%)
**HCM**	96 (49.74%)	25 (12.95%)	12 (6.21%)	5 (2.59%)	2 (1.03%)	52 (26.94%)
**LVNC**	2 (1.03%)	1 (0.51%)	0	1 (0.51%)	0	0
	**193**	**51 (26.42%)**	**20 (10.36%)**	**18 (9.84%)**	**30 (15.54%)**	**74 (38.34%)**
				**122 (63.21%)**		
		**193**				
